# Mechanism of Action against Food Spoilage Yeasts and Bioactivity of *Tasmannia lanceolata*, *Backhousia citriodora* and *Syzygium anisatum* Plant Solvent Extracts

**DOI:** 10.3390/foods7110179

**Published:** 2018-10-29

**Authors:** Fahad Alderees, Ram Mereddy, Dennis Webber, Nilesh Nirmal, Yasmina Sultanbawa

**Affiliations:** 1 Queensland Alliance for Agriculture and Food Innovation, University of Queensland, Brisbane, QLD 4108, Australia; alderees@gmail.com (F.A.); nirmalnp21@yahoo.co.in (N.N.); 2 Department of Agriculture and Fisheries, Brisbane, QLD 4108, Australia; Ram.Mereddy@daf.qld.gov.au (R.M.); Dennis.Webber@daf.qld.gov.au (D.W.)

**Keywords:** natural antimicrobial, antioxidant, mechanism of action, citral, polygodial, anethole

## Abstract

Bioactive properties of solvent extracts of *Tasmannia lanceolata*, *Backhousia citriodora* and *Syzygium anisatum* investigated. The antimicrobial activities evaluated using agar disc diffusion method against two bacteria (*Escherichia coli* and *Staphylococcus aureus*) and six weak-acid resistant yeasts (*Candida albicans*, *Candida krusei*, *Dekkera anomala*, *Rhodotorula mucilaginosa*, *Saccharomyces cerevisiae* and *Schizosaccharomyces pombe*). The antioxidant activities determined using DPPH (2,2-diphenyl-1-picrylhydrazyl) free radical scavenging and reducing power assays. Quantification of major active compounds using ultra-high performance liquid chromatography. Extracts showed broad-spectrum antifungal activity against weak-acid resistant yeasts in comparison to the standard antifungal agents, fluconazole and amphotericin B. *Dekkera anomala* being the most sensitive and strongly inhibited by all extracts, while *Escherichia coli* the least sensitive. Polygodial, citral and anethole are the major bioactive compounds identified in *Tasmannia lanceolata*, *Backhousia citriodora* and *Syzygium anisatum*, respectively. Hexane extracts contain the highest amount of bioactive compounds and demonstrate the strongest antimicrobial activities. Methanol and ethanol extracts reveal the highest phenolic content and antioxidant properties. Fluorescence microscopic results indicate the mechanism of action of *Backhousia citriodora* against yeast is due to damage of the yeast cell membrane through penetration causing swelling and lysis leading to cell death.

## 1. Introduction

The market for soft drinks and fruit juices is increasing annually with the release of new beverage products, which are gaining popularity among consumers. This market expansion has increased the challenge of addressing spoilage problems [1]. Yeasts are the most common group of microorganisms responsible for spoilage of soft drinks and fruit juices due to their ability to withstand juice acidity and resist the action of weak-acid preservatives [1,2]. Beverage industries are focusing on the application of novel antimicrobial agents derived from plant sources as an alternative solution to address beverage spoilage caused by weak-acid resistant yeasts [3]. Tasmanian pepper leaf (*Tasmannia lanceolata*), lemon myrtle (*Backhousia citriodora*) and anise myrtle (*Syzygium anisatum*) are three Australian native herbs in commercial production and there is a growing interest in their bioactive properties and assessing their potential applications as functional ingredients in the beverage industry [4].

Tasmanian pepper leaf belongs to the Winteraceae family and found in forested regions in Tasmania, Victoria [5]. It is used in food as a seasoning, flavoring, coloring and preservative and it is incorporated into personal health care products [4,6,7]. Polygodial is the major bioactive compound in Tasmanian pepper leaf responsible for its strong pungent flavor and reported to be the main contributor to the antibacterial and antifungal activities [4,5,8,9]. A chemical profiling of the essential oil of Tasmanian pepper leaf shows the following sesquiterpene compounds: polygodial (36.74%), guaiol (4.46%), calamenene (3.42%), spathulenol (1.94), drimenol (1.91%), cadina-1,4-diene (1.58%), 5-hydroxycalamenene (1.47%) bicyclogermacrene (1.15%), α-cubebene (0.88%), β-caryophyllene (0.87%), α-copaene (0.48%), cadalene (0.44%), d-cadinol (0.40%), elemol (0.39%), T-muurolol (0.39%) and germacrene-D (0.33%) [10]. Some cultivated Tasmanian pepper leaf clones are found to contain polygodial as high as 64% [10]. Phenolic compounds have been identified as the major class of antioxidant compounds in Tasmanian pepper leaf solvent and polyphenol rich extracts these include coumaric acid, cyanidin-3-glucoside, chlorogenic acid, quercetin, quercetin 3-rutinoside, cyanidin 3-rutinoside [7,11,12].

Lemon myrtle is a member of the Myrtaceae family and mainly grows in the subtropical rainforests of southeast regions of Queensland, Australia [13]. Lemon myrtle contains citral as a predominant compound with antimicrobial and insect repellent properties and is used as a cure for skin diseases [14,15,16]. It has a pleasant lemon flavor with mild sweet notes, it is currently one of the most cultivated and commercialized native plant species utilized in cosmetics, health products, herbal teas and flavoring agents in food and beverage systems [4,17,18,19]. According to reported literature, lemon myrtle essential oil contains citral (82–91%), 5-hepten-2-one,6-methyl (1.54–13.82%), 2,3-dehydro-1,8-cineole (3.52%), nerol (2.66%), germacrene B (0.2–2.18%), geraniol (0.8–1.26%), linalool (0.5–5.85%), myrcene (0.4–4.39%) and citronellal (0.25–2.19%) [15,16,20]. Phenolic compounds detected in solvent and polyphenol rich extracts of lemon myrtle contributing to antioxidant activity were myricetin, hesperetin rhamnoside hesperetin hexoside, quercetin, ellagic acid, ellagitannins and ellagic acid glycosides [11,12].

Anise myrtle also belongs to the Myrtaceae family and grows in subtropical rainforests of Bellingen and Nambucca Valleys of northeast New South Wales and some regions of Queensland [21,22]. Anise myrtle essential oil contains anethole (94.97%), methyl chavicol (4.43%), α-pinene (0.09%), 1,8-cineole (0.02%) and α-farnesene (0.07%) [23]. Anethole possesses antibacterial and antifungal activities and contributes to the intense licorice and aniseed aroma in anise myrtle leaves currently being utilized in cosmetics, savory cuisines, tea blends, body and mouth care products, alcoholic drinks and pharmaceutical industries [22,24,25,26]. Catechin, quercetin, hesperetin, myricetin, ellagic acid, ellagitannins and ellagic acid glycosides were the main antioxidant phenolic compounds in polyphenol rich and solvent extracts of anise myrtle leaves [11,12].

Essential oils of lemon and anise myrtle with citral and anethole as the major volatile compound have shown broad spectrum antimicrobial activity against bacteria, yeast and fungi. The minimum inhibitory concentration (MIC) ranged from 0.16 to >1.25 (% *v*/*v*) for lemon myrtle and 0.63 to >1.25 (% *v*/*v*) for anise myrtle against the following bacteria: *Staphylococcus aureus*, *Escherichia coli*, *Bacillus cereus*, *Proteus vulgaris*, *Pseudomonas aeruginosa*, *Enterobacter aerogenes*, *Acinetobacter baumannii*, *Shewanella putrefaciens* and *Listeria monocytogenes* [27]. For yeast (*Saccharomyces cerevisiae*) and fungi (*Geotricum candidum*) the MIC ranged from 0.04 to 0.08 (% *v*/*v*) for lemon myrtle and 0.16 to 0.08 (% *v*/*v*) anise myrtle respectively [27]. Hexane extracts of lemon myrtle leaves have shown anti-yeast activity against *Candida albicans*, *Candida colliculosa*, *Candida lipolytica*, *Hanseniaspora uvarum*, *Pichia anomala*, *Pichia membranifaciens*, *Rhodotorula mucilaginosa*, *Schizosaccharomyces octosporus*, however, the anise myrtle leaf hexane extracts did not show any activity against these yeasts [28]. Polygodial a major compound in Tasmanian pepper leaf extracts has shown anti yeast activity against *Zygosaccharomyces bailii* and *Saccharomyces cerevisiae*, antifungal activity against *Sclerotinia libertiana*, *Mucor mucedo*, *Rhizopus chinensis*, *Aspergillus niger*, *Penicillium crustosum* and antibacterial activity against *Salmonella choleraesuis* [6,8]. Hexane and methanol extracts of Tasmanian pepper leaf have shown anti-yeast activity against *Candida albicans*, *Candida colliculosa*, *Candida lipolytica*, *Candida stellata*, *Hanseniaspora uvarum*, *Pichia anomala*, *Pichia membranifaciens*, *Rhodotorula mucilaginosa*, *Schizosaccharomyces octosporus* [28]. In the study by Zhao and Agboola [28] water extract had the least antimicrobial activity and hexane was the most potent.

Herb extracts could serve as functional ingredients in soft drinks and fruit beverages due to their antioxidant and antimicrobial properties. In recent years, much attention has been given to the application of natural compounds as an alternative solution to tackle beverage spoilage problems caused by weak-acid resistant yeasts [29,30,31]. The aim of this study is to measure the amounts of major compounds present in different solvent extracts of Tasmanian pepper leaf, lemon myrtle, and anise myrtle and assess the extracts bioactive properties and mechanism of action against weak-acid resistant yeasts and bacteria. This is the first study to assess the antimicrobial properties of different extracts of these herbs against a range of weak-acid resistant spoilage yeasts of importance to the beverage industry.

## 2. Materials and Methods

### 2.1. Plant Material

Lemon myrtle and anise myrtle were supplied by Australian Rainforest Products Pty Ltd. (Lismore, NSW, Australia), and Tasmanian pepper leaf supplied by Diemen Pepper (Birchs Bay, Tasmania, Australia). Herbs received as dried whole leaves and stored at −20 °C until further use.

### 2.2. Milling

Leaves were separated from stems prior to the milling process. The milling was done using a Mixer Mill MM 400 (Retsch, Arzberg, Germany) which utilizes a metal ball inside a stainless-steel grinding jar that vibrates at a high horizontal speed to perform the grinding. Leaves were loaded in jars, sealed and securely positioned on the mixer instrument with a locking mechanism. Leaves were ground into a fine powder for 30 s at a vibrational frequency of 30 Hz. Milling was done on the same day of the experiment and powder used immediately for solvent extraction.

### 2.3. Solvent Extraction

Pressurized liquid extraction method was performed using an accelerated solvent extraction Dionex 350 instrument (Dionex, Sunnyvale, CA, USA). Twenty grams of each milled sample was mixed with 10 g of diatomaceous earth (Thermo-Fisher Scientific, Waltham, MA, USA) and placed in a 100 mL stainless steel extraction cell with a paper filter installed at the bottom end of the cell. Extraction settings were at 60 °C with five cycles under a nitrogen pressure of 1000 psi. Four extraction solvents, ethanol (≥98%) hexane (≥98.5%), methanol (≥98%) and water were used for extraction. Each extract was collected into an amber glass bottle, filtered with No. 1 Whatman filter, transferred into a centrifuge tube and placed in a miVac DUO centrifugal vacuum concentrator (Genevac Ltd., Gardiner, NY, USA) to evaporate the organic solvent at 45 °C. Dried extracts were weighed and stored at 4 °C until further use.

### 2.4. Microorganisms

The antimicrobial activity was assessed on two bacteria, Gram-positive *Staphylococcus aureus* (ATCC 9144) and Gram-negative *Escherichia coli* (ATCC 11775) and six yeasts. The six yeasts comprised of a standard reference strain (ISO TC 34 SC 9 Joint Working Group 5/ISO 11133) *Candida albicans* (ATCC 10231) and weak-acid preservative resistant strains which are *Candida krusei* (ATCC 6258)*, Dekkera anomala* (ATCC 58985), *Rhodotorula mucilaginos*a (ATCC 20129), *Saccharomyces cerevisiae* (ATCC 38555) and *Schizosaccharomyces pombe* (ATCC 26189).

### 2.5. Antimicrobial

All extracted samples were screened for their antimicrobial properties using agar disc diffusion assay. Bacteria grown at 35 °C for 24 h and yeasts at 25 °C for 48 h prior to the day of the experiment. A volume of 100 µL suspension of culture medium (10^5^ cell per mL) adjusted to the appropriate density of 0.5 McFarland standard using a cuvette spectrophotometer at absorbance reading of 540 nm was inoculated on a solid media plate where standard plate count agar (Oxoid, London, UK) was used for bacteria and Sabouraud dextrose agar (Oxoid, London, UK) for yeasts. Dried sample extracts were dissolved in ethanol and 2 mg loaded onto sterile 6 mm paper discs under the fume hood and allowed to dry out before placing on the inoculated plates. Standard antibacterial chloramphenicol (Oxoid, London, UK) at 30 µg/disc and antifungal fluconazole (Sigma-Aldrich, St. Louis, MO, USA) and amphotericin B (Sigma-Aldrich, St. Louis, MO, USA) at 20 µg/disc were included as positive controls. Ethanol and water used to dissolve the standard antimicrobial drugs and assayed as negative controls. Incubation was done at 35 °C for 24 h for bacteria and at 25 °C for 48 h for yeasts. The experiments were done in duplicate. Zones of inhibition were measured using a digital caliper and expressed in millimeters. Criteria for antimicrobial strength were divided into three ranges according to Ahmad et al. [32]: weak activity (inhibition zone <10 mm), moderate activity (inhibition zone 10 to 15 mm) and strong activity (inhibition zone >15 mm).

### 2.6. Yeast Cell Staining and Fluorescence Microscopy

Yeast cells, *S. cerevisiae*, were grown at 25 °C for 24 h in tryptone soya yeast extract broth (Oxoid, London, UK). Yeast cells suspension was adjusted to the appropriate density of 0.5 McFarland standard using a cuvette spectrophotometer at absorbance reading of 540 nm. The adjusted suspension divided into control and treatment groups. The treatment group was centrifuged at 2500 rpm for 3 min, supernatant was removed, 4% (*v*/*v*) lemon myrtle hexane extract (dissolved in sterile water containing 0.4% tween-80) was added and allowed to stand for 30 and 60 min. The control group treated in the same manner except 0.1 M phosphate buffer added instead of lemon myrtle extract. Suspensions of treatment and control groups centrifuged at 2500 rpm for 3 min and washed with 0.1 M phosphate buffer. Cells fixed and stained according to Shimada et al. [33] with few modifications. Cells fixed for 30 min by the addition of 4% paraformaldehyde (4 mL), centrifuged (2500 rpm, 3 min) and washed twice with 0.1 M phosphate buffer. Cell suspension was mixed with 1:1 *v*/*v* of 50 ng/mL DAPI (4′,6-diamidino-2-phenylindole) (Thermo-Fisher Scientific, Waltham, MA, USA) and 8 µL of the mixture was added on a glass microscope slide and covered with a coverslip. Cell fluorescence images observed using a Leica DM6000B microscope with Leica Microsystem LAS AF6000 software (Leica, Hamburg, Germany) at 100× objective using a Leica DFC420C digital camera (Leica, Hamburg, Germany).

### 2.7. Total Phenolic Content

Total phenolic content (TPC) of ethanol, hexane, methanol and water extracted herb samples were spectrophotometrically analyzed according to Folin–Ciocalteu colorimetric technique [34]. Samples were diluted (10–1000 times) with Mili-Q water and 25 µL from each dilution added to the 96-well polystyrene plate (Sarstedt, Nümbrecht, Germany). Several concentrations of gallic acid (3,4,5-Trihydroxybenzoic acid ≥98%, Sigma-Aldrich, St. Louis, MO, USA), 0–100 mg/L, were prepared to construct the standard calibration curve and 25 µL of gallic acid was loaded into the plate. All wells were loaded with 125 µL of Folin-Ciocalteu’s phenol reagent (Sigma-Aldrich, St. Louis, MO, USA), followed by the addition of 125 µL Sodium carbonate (Chem-Supply, Gillman, Australia). The 96-well plate placed in a microplate-reader (Tecan, Grödig, Austria) and shaken for 15 s and the absorbance reading measured at 750 nm after 15 min of incubation in the dark. Calculated results expressed as milligram of gallic acid equivalents per gram of sample dry weight (GAE/g DW).

### 2.8. DPPH Radical Scavenging Activity

The radical scavenging activity of the ethanol, hexane, methanol and water extracted herb samples were evaluated using DPPH (2,2-diphenyl-1-picrylhydrazyl) free radical scavenging assay according to Nirmal and Panichayupakaranant [32] with slight modifications. On the day of the experiment, DPPH (Sigma-Aldrich, St. Louis, MO, USA) concentration of 0.15 mM was prepared in 95% ethanol and mixed with different sample concentrations at 1:1 ratio (*v*/*v*) in a total volume of 3 mL. All reagents brought to room temperature before mixing. The mixing was carried out in the dark at room temperature for 30 min and the absorbance measured at 517 nm using a cuvette spectrophotometer (Thermo-Fisher Scientific, Waltham, MA, USA). Sample blank was prepared in the same manner except ethanol was used instead of DPPH solution, while control included DPPH solution and ethanol but without the addition of any sample. The percentage inhibition capacity of scavenging property for the DPPH radicals was calculated using the following formula:

% inhibition = (1 − (sample absorbance/control absorbance)) × 100.(1)

### 2.9. Reducing Power

The reducing power of herb extracts analyzed as described by Nirmal and Panichayupakaranant [35]. Different concentrations of leaf extracts were prepared in a phosphate buffer (0.2 M, pH 6.6, 1 mL), mixed with 1 mL of 1% potassium ferric cyanide (Sigma-Aldrich, St. Louis, MO, USA) and allowed to incubate at 50 °C for 20 min. One milliliter of 10% trichloroacetic acid (Sigma-Aldrich, St. Louis, MO, USA) added to the mixture and centrifuged at 2500 rpm for 10 min. One milliliter from the top layer of the solution was removed and mixed with 1 ml distilled water and 0.2 mL of 0.1% ferric chloride (Sigma-Aldrich, St. Louis, MO, USA) and absorbance measured at 700 nm using a cuvette spectrophotometer. The strength of reducing power was indicated by the higher absorbance reading.

### 2.10. UHPLC-MS Analysis

The quantification of polygodial, citral and anethole, major compounds found in the essential oil of the tested herbs was performed by UHPLC-MS (ultra-high performance liquid chromatography/mass spectrometry) system. Extracts dissolved in methanol (HPLC Grade, Merck, Darmstadt, Germany) for analysis. The system consisted of Dionex UlitMate 3000 (Thermo-Fisher Scientific, Waltham, MA, USA) equipped with a quaternary solvent delivery system coupled to a Thermo-Fisher Q Exactive High Resolution Accurate Mass MS (Thermo-Fisher Scientific, Waltham, MA, USA). The instrument was fitted with an Acquity UHPLC BEH Shield 1.7 µm, 2.1 × 100 mm column (Waters, Milford, MA, USA). Chromatographic separation carried out with mobile phase A (Mili-Q water containing 0.1% formic acid) and mobile phase B (acetonitrile containing 0.1% formic acid). The linear gradient program was as follows: 0–0.3 min, 5% B; 0.3–2 min, 5–25% B, 2–3.5 min 25–50%, 3.5–4 min 50–80%, 4–7.5 min 80%, 7.5–8 min 80–5% and 8–11 min 5%. The flow rate was 0.5 mL/min, the column temperature was 40 °C, the autosampler temperature was 15 °C and the injection volume was 1 µL. The UHPLC-MS run in ESI-positive ion mode. Parameters were as follows: capillary temperature 268 °C; auxiliary gas heater temperature 438 °C; spray voltage 3.5 kV; sheath gas flow rate 52; auxiliary gas flow rate 14; sweep gas flow rate 3; and S-lens RF level 50. Data was collected from 3 to 10 min, over a mass range of 50–300 *m*/*z*, with a maximum IT of 200 ms and a resolution setting of 70,000 at 200 *m*/*z*. Quantification of analytes performed by external calibration, using known standard solutions of polygodial (Sigma-Aldrich, St. Louis, MO, USA), citral (Sigma-Aldrich, St. Louis, MO, USA) and anethole (Sigma-Aldrich, St. Louis, MO, USA).

### 2.11. Statistical Analysis

Results were expressed as mean ± standard deviation and all statistical analyses were performed using GraphPad Prism version 7.00 (GraphPad 2016, Version 7.03, GraphPad Software, Inc., San Diego, CA, USA) and figures were generated in Microsoft Excel (Office 2016, Microsoft, Redmond, WA, USA). Statistical significance of differences among treatment groups done by using one-way analysis of variance (ANOVA) followed by Tukey’s test as a post hoc comparison and *p* < 0.05 is considered significant. Pearson’s correlation coefficient was used to determine correlation between total phenolic content and antioxidant capacities.

## 3. Results

### 3.1. Extraction Yield and Extracts Characteristics

Yield of extractable compounds obtained from using different solvents evaluated and presented in Table 1. In general, the ranking of extraction yields obtained from different solvents are as follows in decreasing order: methanol > ethanol > water > hexane. There was a significant difference in yield between solvents used in extraction except for methanolic and ethanolic extracts of Tasmanian pepper leaf and ethanolic and water extracts of anise myrtle.

### 3.2. Total Phenolic Content

The TPC of all herbs from different solvent extracts shown in Table 1. Results of the total extractable phenols expressed as mg gallic acid equivalent (GAE) per g of sample dry weight. Lemon myrtle possessed the highest TPC followed by anise myrtle and Tasmanian pepper leaf. The extraction of phenolic compounds varied among solvents used in this experiment, methanol extracts had the highest phenols followed by ethanol, water and hexane.

### 3.3. DPPH Radical Scavenging Activity

The free radical scavenging capacity of herb extracts from different solvents given in Table 1. The results expressed in IC_50_ (half-maximal inhibitory concentration) values are interpreted as the concentration of antioxidants required to reduce the free radical DPPH by half in the solution; lower IC_50_ values represent strong free radical scavenging activity. The strongest scavenging capacity are shown in the extracts of lemon myrtle, followed by anise myrtle and Tasmanian pepper leaf. The radical scavenging activities of extracts significantly varied with different solvents and ranked in decreasing order as methanol = ethanol > water > hexane.

### 3.4. Reducing Power

The reducing potential of a substance reflects its antioxidant capacity measured by utilizing the reducing power assay. The presence of antioxidant compounds (reductants) in the tested herb extracts, will react with the potassium ferricyanide (Fe^3+^) reducing it into potassium ferrocyanide (Fe^2+^) causing color transformation of the assay solution from yellow into different color spectrums of green and yellow which can be measured at 700 nm. Table 1 shows the reducing power of herbs extracted from different solvents. The reducing power ranking of herb extracts follows the same pattern (lemon myrtle > anise myrtle > Tasmanian pepper leaf) as the previous two assays, DPPH and TPC. The extracts reducing power potential is concentration dependent; it increases as extract concentration increases. Methanol and ethanol extracts had the highest reducing power, followed by water extracts having a moderate reducing potential while hexane extracts showed the least reducing capacity.

### 3.5. Relationship between Total Phenolic Content and Antioxidant Capacities

**Figure 1:**
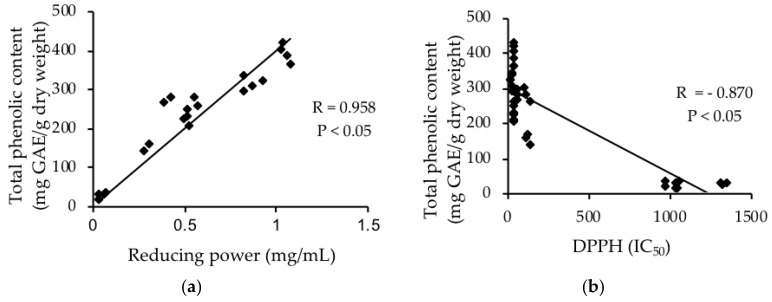
Correlation between total phenolic content and antioxidant capacities measured by DPPH and reducing power in Australian native herbs. (**a**) Correlation between reducing power and total phenolic content; (**b**) Correlation between DPPH and total phenolic content.

The antioxidant capacities of all tested herb extracts measured by the two assays DPPH and reducing power were found to have a significant linear correlation with the TPC. A regression analysis, correlation coefficient, performed to correlate TPC with reducing power (Figure 1a) and TPC with DPPH (Figure 1b). The overall correlation coefficient (R) between TPC and DPPH was −0.870 and between TPC and reducing power was 0.958. The correlation coefficient (R) values between TPC and DPPH were −0.988, −0.966 and −0.939 and between TPC and reducing power were 0.907, 0.979 and 0.983 for anise myrtle, lemon myrtle and Tasmanian pepper leaf, respectively. The strong relationship between TPC and antioxidant results is a clear indication that the phenolic compounds of the herb extracts contributed to the antioxidant capacity.

### 3.6. Antimicrobial Activities

The mean values and standard deviations for inhibition zones of herb extracts from various solvents and standard antimicrobial drugs against yeasts and bacteria, using agar disc diffusion method, are given in Table 2. The herb extracts from different solvents showed varied antimicrobial activities against the tested microorganisms. Hexane extracts produced the largest inhibition zones showing strong antifungal activity (inhibition zone > 15 mm) in most weak-acid resistant yeasts, followed by methanol and ethanol extracts, while water extracts showed no activity against all tested microorganisms. Extracts from hexane, methanol and ethanol showed broad-spectrum antimicrobial activity against tested microorganisms showing moderate activity (inhibition zone 10 to 15 mm) and strong activity, except for the methanolic and ethanolic extracts of lemon myrtle and anise myrtle, which did not show inhibition against *E. coli*. In addition, the hexane extract of anise myrtle showed no inhibition against *E. coli.* The standard fluconazole had broad-spectrum activity against all tested yeasts showing moderate and strong activity, while amphotericin B had a narrow-spectrum that only inhibited *D. anomala*, *S. pombe* and *R. mucilaginosa*, with inhibition zones between medium and strong activity*.* All hexane extracts had significantly higher zones of inhibition in comparison to fluconazole and amphotericin B at the tested concentrations, except against *D. anomala* for Tasmanian pepper leaf and anise myrtle. Furthermore, methanol and ethanol extracts of lemon myrtle and anise myrtle exhibited comparable inhibition zones to fluconazole and amphotericin B. In the case of bacteria, chloramphenicol showed significantly higher zones of inhibition than herb extracts. The negative controls, ethanol and water, produced no zones of inhibition.

### 3.7. Mode of Antifungal Action

**Figure 2:**
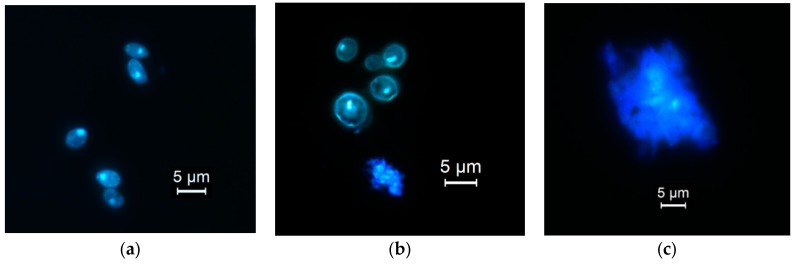
Illustration of morphological changes to yeast cells of *S. cerevisiae* at different treatment stages. (**a**) Untreated cells having a normal oval-shape; (**b**) Lemon myrtle extract (4% *v*/*v*) cell treated for 30 min showing swollen round-shaped cells next to a membrane damaged cell and (**c**) treatment for 60 min exhibits clear cell membrane rupture.

Lemon myrtle hexane extract showed the most effective anti-yeast activity and selected to investigate the antifungal mechanism of action against *S. cerevisiae. S. cerevisiae* is a widely studied eukaryotic model yeast with a relatively large cell size for better observation and investigation for morphological changes [36]. The mechanism of antifungal action of hydrophobic bioactive compounds is reported as penetrating and damaging cellular cytoplasmic membrane [37,38]. Images of *S. cerevisiae* untreated, 30 min and 60 min treated cells are given in Figure 2. The untreated cells appear to have a normal elongated-oval shape with no signs of deformation or damage to cell structure (Figure 2a). Cells exposed to treatment for a period of 30 min have undergone structural modification where cells became swollen and changed from being oval into a circular shape; in addition, in some cells the membrane collapsed and reduced in size (Figure 2b). When treatment increased to 60 min, cell damage became more profound showing cells with ruptured membrane (Figure 2c). The DAPI staining observed to be brighter on damaged cells compared to non-damaged cells this could be due to the stain accumulating on the rough layer of damaged cell membrane. It was also noticeable when cells were exposed to the fluorescence microscope light source for a longer period, the stain started to fade away at a faster rate only in membrane ruptured cells. This phenomenon indicates that the stain is contained within the boundary of non-ruptured cells and is protected inside the cell and requires a longer light exposure time to fade away compared to the stain that has leaked out of the damaged cells.

### 3.8. UHPLC-MS Analysis of Herb Extracts

**Figure 3:**
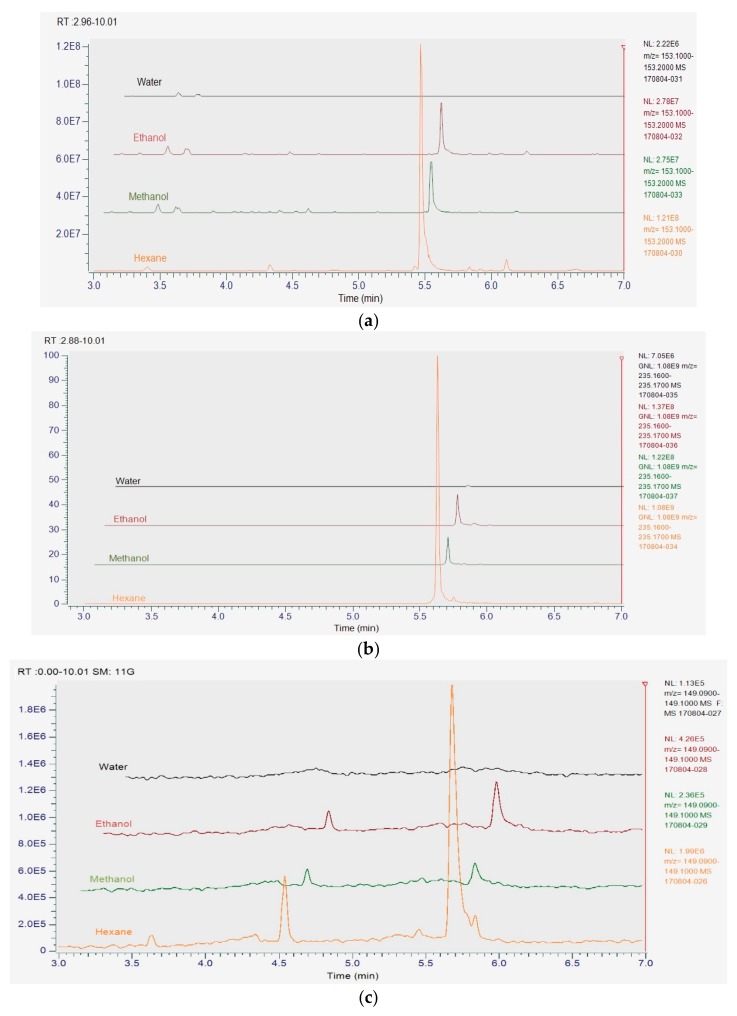
Chromatogram of major compounds extracted from Australian native herbs in different solvents. Citral in lemon myrtle extracts (**a**); polygodial in Tasmanian pepper leaf extracts (**b**) and anethole in anise myrtle extracts (**c**).

UHPLC-MS analysis showed hexane extracts contained the highest concentration of major compounds, polygodial, citral and anethole, whereas no detectable readings were found in water extracts due to the hydrophobic characteristic of these compounds. Methanol and ethanol extracts indicate similar concentration of major compounds, which were significantly lower (*p* < 0.05) than the hexane extracts (Figure 3). The major compounds identified in herb extracts and their extracted quantities are listed as follows: polygodial found in Tasmanian pepper leaf at 41.70%, 4.43% and 3.91%; citral found in lemon myrtle at 45.7%, 9.62% and 9.29%; and anethole found in anise myrtle at 37.1%, 1.80% and 4.16%; from hexane, ethanol and methanol, respectively.

## 4. Discussion

Type of solvents used in this study had an impact on antimicrobial and antioxidant properties of herb extracts. Variation in solvent polarity is the key for the different concentrations of extracted active compounds [39]. Results showed that hexane extracts had higher antimicrobial activities compared with ethanol and methanol extracts of Tasmanian pepper leaf, lemon myrtle and anise myrtle, whereas their aqueous extracts did not exhibit antimicrobial activity. This indicates that nonpolar compounds had contributed to the antimicrobial activity of these three herbs. Previous reports found that the nonpolar compounds, polygodial, citral and anethole, were the dominant compounds in the essential oil of Tasmanian pepper leaf, lemon myrtle and anise myrtle, respectively, which could be the main contributors to the reported antibacterial and antifungal property of these herbs [10,23,24,40,41,42]. The antimicrobial activity of plant essential oils is often attributed to the main compounds; however, the minor compounds could contribute to antimicrobial activity and may work in synergy by forming a complex interaction with the major compounds enhancing their antimicrobial action [43,44]. For example, a study done by Sultanbawa et al. [45] evaluated the antimicrobial activity of lemon myrtle essential oil and its major bioactive compound citral against *S. aureus* and *E. coli*. The minimum inhibitory concentration of citral is 4-fold and 8-fold higher compared to the essential oil of lemon myrtle against *S. aureus* and *E. coli*, respectively.

Hexane extracts showed higher antimicrobial activity but performed poorly in the antioxidant aspect since majority of the antioxidant phenolic compounds are polar and not readily soluble in nonpolar solvents. Herb phenolic compounds have been reported to be efficiently extracted in solvents with higher polarity which makes water a superior solvent for the extraction of antioxidant compounds [46,47,48]. However, this was not the case in the current study since methanol and ethanol extracts contained the highest phenolic content and exerted strongest antioxidant activity, which shows the presence of some lipophilic antioxidant compounds in these herbs. Konczak et al. [12] reported that lipophilic fraction made a significant contribution to the antioxidant activity in lemon myrtle at 45.8% and anise myrtle, Tasmanian pepper leaf to a lesser degree at 5% and 14% respectively. Ellagic acid and its derivatives have been identified as the main phenolic compounds in lemon myrtle and anise myrtle extracts, while chlorogenic acid and quercetin in Tasmanian pepper leaf extract [49]. Therefore, selectivity of suitable solvents and extraction methods like ultrasonically or microwave assisted extractions for extracting phenolic compounds is important due to the diverse composition of phytochemicals in botanicals and differences in their lipophilic and hydrophilic characteristics [50,51].

The methanolic extracts of lemon myrtle, anise myrtle and Tasmanian pepper leaf showed higher TPC by 13.4, 5.6 and 2.4 fold, respectively, in comparison to the findings of Konczak et al. [12]. In addition, they reported that lemon myrtle extracts have the least antioxidant activity and phenolic content compared to anise myrtle and Tasmanian pepper leaf extracts. On the contrary, we found lemon myrtle extracts to possess the highest antioxidant activity and phenolic content. Increases in the reported TPC may be due to extraction conditions, which were done under a nitrogen pressure of 1000 psi combined with temperature of 60 °C and five extraction cycles in an accelerated solvent extraction instrument. Konczak et al. [12] sonicated herb samples for 10 min in an aqueous acidified methanol (19% water, 80% methanol, 1% hydrochloric acid) under a nitrogen atmosphere with a total of three extraction cycles. Such differences in extraction conditions between studies could have influenced the extraction and solubility of phenolic compounds [52]. Konczak et al. [12] also found a good correlation between the levels of total polyphenol content (mg GAE/g DW) and the reducing power antioxidant assays at *R*^2^ = 0.8315 for native Australian herbs and spices which is comparable to this study. Seasonality and time of harvest are reported to significantly influence the phenolic compound content in plants, which could be another possible reason for differences between results [53].

Differences between extraction methods not only could influence the phenolic content and antioxidant activity of herbs, but also affect their antimicrobial activity. In general, direct comparison between studies of antimicrobial activity of herb extracts is challenging due to variation in methodologies including extraction conditions for bioactive compounds, and extraction concentrations. In a previous study anise myrtle extracts at a concentration of 10 mg per disc did not show any activity against *S. aureus*, *C. albicans* and *R. mucilaginosa* [28]. However, in our study anise myrtle extracts of 2 mg per disc showed moderate to strong inhibition against these microorganisms. In addition, the study found no activity against *S. aureus* and *C. albicans* from lemon myrtle methanol extract (10 mg per disc) and no activity against *E. coli* from lemon myrtle hexane extract (10 mg per disc)*,* which is the opposite of our findings where lemon myrtle (2 mg per disc) showed antimicrobial activity against these microorganisms. In the study by Zhao and Agboola [28] the herb extractions were performed using a magnetic stirrer for 30 min at room temperature, which may not be an efficient method for the extraction of bioactive compounds. Results of extraction solvent were in agreement with Zhao and Agboola [28] in which methanol is the most efficient solvent in extracting antioxidant phenolic compounds and produced the highest extraction yield, while hexane is the weakest in both antioxidant capability and extraction yield. In addition, a similar trend in DPPH free radical scavenging capacity is supported as lemon myrtle being the strongest, followed by anise myrtle and Tasmanian pepper leaf.

Herb extracts demonstrated an overall moderate to strong antifungal activity in comparison to the standard antifungal drugs against tested yeasts. Our antibacterial results agreed with many published studies in which Gram-positive *S. aureus* is more sensitive to herb extracts than Gram-negative *E. coli* [46,54,55,56].

The current study showed that lemon myrtle extracts had executed their action through targeting yeast membrane in a time-dependent fashion. This observation is confirmed by Bakkali et al. [37] and Chuang et al. [57] reporting on time being required for the active compounds to partition themselves into the cell membrane and gain entry to the cell causing the observed size enlargement and swelling to the cell structure which eventually lead to rupture of cell membrane. Few studies have reported the ability of essential oils to cause swelling to bacteria, fungi and protozoa cells due to their hydrophobic properties [58,59,60]. Another explanation to yeast cell membrane lysis is through the binding and interacting with ergosterol found on cell membrane. Ergosterol is the main sterol component of fungal cell membrane which is responsible for the membrane rigidity, fluidity, and permeability. Therefore, binding to the ergosterol in cell membrane causes expanding of membrane lipid bilayer, altering membrane permeability and forming channels which are responsible for the cell cytoplasmic content leakage and eventual cell death [61,62]. Mechanism of action of citral, found in lemon myrtle extracts, was reported to be through the binding with ergosterol leading to membrane functional and structural destabilization [63].

## 5. Conclusions

In conclusion, this study reports on the antifungal activity of *Tasmannia lanceolata*, *Backhousia citriodora* and *Syzygium anisatum* extracts against weak-acid resistant yeasts and elucidates the anti-yeast mode of action of Backhousia citriodora extracts. The current results suggest that these three Australian native herbs possess the ability to inhibit the growth of food-spoilage yeasts that are resistant to organic weak-acids, which suggests the potential application in food and beverage industries as an alternative to synthetic antimicrobial agents.

## Figures and Tables

**Table 1:** Yields, total phenolic content, DPPH free radical scavenging and reducing power of Tasmanian pepper leaf, lemon myrtle and anise myrtle extracts.

			Methanol	Ethanol	Water	Hexane
Yields (% *w*/*w*)		LM	22.8 ± 0.4 ^a^	17.9 ± 0.5 ^b^	16.3 ± 0.6 ^c^	6.41 ± 0.2 ^d^
		TPL	28.3 ± 0.3 ^a^	27.8 ± 0.4 ^a^	25.8 ± 0.3 ^b^	8.13 ± 0.5 ^c^
		AM	21.8 ± 0.4 ^a^	16.8 ± 0.6 ^b^	17.5 ± 0.7 ^b^	3.88 ± 0.3 ^c^
Total phenolic content (mg GAE/gDW)		LM	419.3 ± 13.5 ^a^	373.2 ± 12.6 ^b^	281.7 ± 21.6 ^c^	17.5 ± 1.7 ^d^
		TPL	246.3 ± 17.4 ^a^	215.5 ± 12.8 ^a^	157.4 ± 14.6 ^b^	35.7 ± 1.9 ^d^
		AM	314.2 ± 17.3 ^a^	310.6 ± 18.3 ^a^	283.3 ± 16.5 ^b^	30.5 ± 2.1 ^c^
DPPH (IC_50_ µg/mL)		LM	14.4 ± 0.4 ^a^	14.3 ± 0.6 ^a^	31.0 ± 1.1 ^b^	1678.3 ± 27.9 ^c^
		TPL	36.9 ± 0.6 ^a^	36.2 ± 0.8 ^a^	126.4 ± 16.1 ^b^	1004.7 ± 35.9 ^c^
		AM	19.1 ± 1.2 ^a^	21.1 ± 0.1 ^a^	61.9 ± 0.2 ^b^	1342.7 ± 22.9 ^c^
Reducing power (Absorbance 700 nm)	0.01 mg/mL extracts	LM	0.59 ± 0.01 ^a^	0.59 ± 0.02 ^a^	0.32 ± 0.01 ^b^	0.03 ± 0.01 ^c^
		TPL	0.29 ± 0.01 ^a^	0.31 ± 0.01 ^a^	0.14 ± 0.01 ^b^	0.04 ± 0.02 ^c^
		AM	0.49 ± 0.02 ^a^	0.45 ± 0.02 ^a^	0.25 ± 0.01 ^b^	0.025 ± 0.01 ^c^
	0.1 mg/mL extracts	LM	1.03 ± 0.01 ^a^	1.07 ± 0.02 ^a^	0.56 ± 0.02 ^b^	0.03 ± 0.01 ^c^
		TPL	0.51 ± 0.01 ^a^	0.52 ± 0.01 ^a^	0.30 ± 0.02 ^b^	0.07 ± 0.01 ^c^
		AM	0.87 ± 0.1 ^a^	0.84 ± 0.03 ^a^	0.41 ± 0.03 ^b^	0.03 ± 0.01 ^c^

DPPH: 2,2-diphenyl-1-picrylhydrazyl; LM: lemon myrtle; TPL: Tasmanian pepper leaf; AM: anise myrtle; mg GAE/gDW: milligram gallic acid equivalents/g dry weight. Reducing power results expressed from testing concentrations of 0.01 and 0.1 mg/mL of extracts. Means with different letters within the same row are significantly different at *p* < 0.05.

**Table 2:** Inhibition zone (mm) of Australian native herb extracts from hexane, methanol and ethanol against yeasts and bacteria.

		*D. anomala*	*S. pombe*	*S. cerevisiae*	*C. albicans*	*R. mucilaginosa*	*C. krusei*	*S. aureus*	*E. coli*
TPL	M	17.6 ± 0.8 ^b^	13.7 ± 0.3 ^b^	17.2 ±0.5 ^b^	14.2 ± 0.3 ^b^	17.1 ± 0.8 ^b^	16.4 ± 0.5 ^c^	12.3 ± 0.4 ^b^	9.0 ± 0.2 ^b^
	E	16.4 ± 0.6 ^c^	13.0 ± 0.3 ^b^	17.4 ± 0.4 ^b^	14.8 ± 0.3 ^b^	16.7 ± 0.4 ^b^	15.2 ± 0.7 ^b^	12.2 ± 0.2 ^b^	8.3 ± 0.4 ^b^
	H	23.9 ± 0.4 ^a^	17.0 ± 0.3 ^a^	20.7 ± 0.4 ^a^	17.1 ± 0.4 ^a^	21.4 ± 0.7 ^a^	19.6 ± 0.5 ^a^	13.6 ± 0.2 ^a^	10.9 ± 0.3 ^a^
LM	M	27.3 ± 0.7 ^b^	12.1 ± 0.8 ^b^	11.8 ± 1.2 ^b^	12.7 ± 1.3 ^b^	14.7 ± 0.9 ^b^	11.0 ± 0.8 ^b^	10.1 ± 0.4 ^a,b^	0
	E	24.1 ± 0.9 ^c^	11.1 ± 0.5 ^b^	10.9 ± 1.0 ^b^	14.1 ± 0.7 ^b^	14.1 ± 0.7 ^b^	10.1 ± 0.7 ^b^	9.3 ± 0.7 ^b^	0
	H	43.3 ± 2.1 ^a^	35.7 ± 1.2 ^a^	34.9 ± 1.4 ^a^	26.8 ± 0.7 ^a^	21.04 ± 1.8 ^a^	19.8 ± 1.4 ^a^	11.5 ± 0.8 ^a^	8.2 ± 0.7 ^a^
AM	M	23.4 ± 0.5 ^b^	13.3 ± 0.7 ^b^	11.3 ± 0.6 ^b^	13.1 ± 0.6 ^b^	14.9 ± 0.3 ^b^	10.0 ± 0.3 ^b^	11.4 ± 0.5 ^a^	0
	E	21.9 ± 0.8 ^c^	10.5 ± 0.7 ^c^	9.9 ± 0.7 ^c^	10.9 ± 0.9 ^c^	14.2 ± 0.4 ^b^	8.9 ± 0.3 ^c^	8.8 ± 0.5 ^b^	0
	H	26.9 ± 0.4 ^a^	14.7 ± 0.4 ^a^	13.2 ± 0.5 ^a^	14.7 ± 0.7 ^a^	16.9 ± 0.6 ^a^	11.3 ± 0.8 ^a^	12.5 ± 0.6 ^a^	0
Fluconazole		37.2 ± 4.5	11.1 ± 0.3	11.4 ± 0.5	10.9 ± 0.9	9.5 ± 1.5	12.7 ± 0.3	NT	NT
Amphotericin B		18.4 ± 0.3	11.9 ± 0.4	0	0	12.8 ± 0.8	0	NT	NT
Chloramphenicol		NT	NT	NT	NT	NT	NT	24.0 ± 1.9	20.5 ± 0.6

TPL: Tasmanian pepper leaf; LM: lemon myrtle; AM: anise myrtle; M: methanol; E: ethanol; H: hexane; NT: not tested. Extract concentration of 2 mg/6 mm disc. Positive controls: 30 µg/6 mm disc chloramphenicol against bacteria, 20 µg/6 mm disc fluconazole and amphotericin B against yeasts. Columns sharing different letters within the same herb extract treatment are significantly different at *p* < 0.05. ^a^ Tasmanian pepper leaf; ^b^ lemon myrtle and ^c^ anise myrtle. Water extracts showed no inhibition. Criteria for antimicrobial activity: <10 mm, weak; 10–15 mm, moderate; >15 mm, strong.
